# Mineralisation of collagen rich soft tissues and osteocyte lacunae in *Enpp1^−/−^* mice

**DOI:** 10.1016/j.bone.2014.09.016

**Published:** 2014-12

**Authors:** Mark O.R. Hajjawi, Vicky E. MacRae, Carmen Huesa, Alan Boyde, José Luis Millán, Timothy R. Arnett, Isabel R. Orriss

**Affiliations:** a Department of Cell and Developmental Biology, University College London, London, UK; b The Roslin Institute and Royal (Dick) School of Veterinary Studies, University of Edinburgh, Edinburgh, UK; c Institute of Dentistry, Bart's and the London School of Medicine and Dentistry, Queen Mary, University of London, UK; d Sanford-Burnham Medical Research Institute, La Jolla, CA, USA; e Department of Comparative Biomedical Sciences, Royal Veterinary College, London, UK

**Keywords:** NPP1, Osteocytes, Osteoclasts, Soft tissue mineralisation, Pyrophosphate

## Abstract

Ecto-nucleotide pyrophosphatase/phosphodiesterases (NPPs) hydrolyse nucleotide triphosphates to the corresponding nucleotide monophosphates and the mineralisation inhibitor, pyrophosphate (PP_i_). This study examined the role of NPP1 in osteocytes, osteoclasts and cortical bone, using a mouse model lacking NPP1 (*Enpp1^−/−^*). We used microcomputed tomography (μCT) to investigate how NPP1 deletion affects cortical bone structure; excised humerus bones from 8, 15 and 22-week old mice were scanned at 0.9 μm. Although no changes were evident in the cortical bone of 8-week old *Enpp1^−/−^* mice, significant differences were observed in older animals. Cortical bone volume was decreased 28% in 22-week *Enpp1^−/−^* mice, whilst cortical porosity was reduced 30% and 60% at 15 and 22-weeks, respectively. This was accompanied by up to a 15% decrease in closed pore diameter and a 55% reduction in the number of pores. Cortical thickness was reduced up to 35% in 15 and 22-week *Enpp1^−/−^* animals and the endosteal diameter was increased up to 23%. Thus, the cortical bone from *Enpp1^−/−^* mice was thinner and less porous, with a larger marrow space. Scanning electron microscopy (SEM) revealed a decrease in the size and number of blood vessel channels in the cortical bone as well as a 40% reduction in the mean plan area of osteocyte lacunae. We noted that the number of viable osteocytes isolated from the long bones of *Enpp1^−/−^* mice was decreased ≤ 50%. In contrast, osteoclast formation and resorptive activity were unaffected by NPP1 deletion. μCT and histological analysis of *Enpp1^−/−^* mice also revealed calcification of the joints and vertebrae as well as soft tissues including the whisker follicles, ear pinna and trachea. This calcification worsened as the animals aged. Together, these data highlight the key role of NPP1 in regulating calcification of both soft and skeletal tissues.

## Highlights

• We examine the role of NPP1 in osteocytes, osteoclasts and cortical bone.
• Osteocyte lacunae are reduced in size and number in NPP1 knockout mice.
• Sclerostin levels are increased in NPP1 knockout mice.
• Osteoclast formation and resorptive activity are unaffected by NPP1 deletion.
• Mice lacking NPP1 display widespread calcification of collagen rich soft tissues.

## Introduction

The ability of pyrophosphate (PP_i_) to inhibit mineralisation was first identified in the 1960s by Fleisch and colleagues [1]. It is now accepted that the ratio of PP_i_ to phosphate (P_i_) within the bone microenvironment is a fundamental regulator of the level of mineralisation [2], [3]. PP_i_ can be generated from nucleotide triphosphates (such as ATP or UTP) by the actions of ecto-nucleotidase pyrophosphatase/phosphodiesterases (NPPs). These transmembrane proteins display widespread expression and are highly conserved between species. Osteoblasts express at least 3 members of the NPP family (NPP1, 2, 3) [4], [5], [6], and of these, NPP1 (previously called plasma cell membrane glycoprotein 1 or PC-1), is thought to be the most important NPP in PP_i_ generation. Tissue-nonspecific alkaline phosphatase (TNAP) is the key enzyme involved in PP_i_ breakdown [4]. Previous work has shown that the opposing actions of NPP1 and TNAP are critical in determining the extracellular P_i_/PP_i_ ratio and, thus, the level of skeletal mineralisation [5]. Furthermore, the inhibitory actions of ATP and UTP on bone mineralisation appear to be partly mediated *via* the actions of NPP1 [6], [7], [8].

NPP1 expression has now been demonstrated in a wide range of tissues and cell types including the heart, kidney, vascular smooth muscle cells and chondrocytes [9], [10], [11], [12]. In addition to its role in regulating mineralisation, NPP1 is also thought to be involved in glucose homeostasis and insulin signalling [13], [14], [15]. Mutations in the gene encoding NPP1 (*Enpp1*) have been associated with a rare autosomal recessive disease generalised arterial calcification of infancy (GACI) [16]. Sufferers of this condition usually die in infancy because of substantial vascular calcification.

The important role of NPP1 in the regulation of tissue mineralisation has been highlighted by two different mouse models; the naturally occurring NPP1 ‘knockout’ termed the tip-toe walking (*ttw/ttw*) mouse and the genetically altered NPP1 knockout (*Enpp1^−/−^*). The *ttw/ttw* model displays ossification of the spinal ligaments, peripheral joint hyperstosis and calcification of articular cartilage [17]. The phenotype of *ttw*/*ttw* mice also has similarities to the human disease ‘Ossification of the posterior longitudinal ligament of the spine’ (OPLL). The *Enpp1^−/−^* model displays a number of defects related to reduced PP_i_ production, including calcification of the aorta, kidney, spine, joints and cartilage [4], [18], [19], [20], [21]. Suprisingly, given the reduction in extracellular PP_i_ levels, *Enpp1^−/−^* mice display reduced trabecular and cortical bone in the long bones and decreased bone strength [20]. These animals also have increased FGF-23 levels [20] and decreased osteopontin levels [19]. Earlier work also showed an increased number of osteoclasts on the *ex vivo* bone surfaces of *Enpp1*^−/−^ mice [20]. This was associated with an increase in the blood serum concentration of the bone resorption marker, C-terminal telopeptides of type I collagen (CTx), and suggests increased osteoclast activity in *Enpp1*^−/−^ animals [20].

Osteocytes, the most abundant cell type in bone [22], reside within lacunae surrounded by mineralised matrix. Extensive dendritic processes extend through canaliculi to form a highly connected communication network between osteocytes and the other cells on the bone surface. Soluble factors released by osteocytes regulate osteoblast and osteoclast function thereby providing a mechanism to control bone remodelling [23], [24]. Osteocytes also play a role in phosphate metabolism, calcium availability and are the primary mechanosensory cell within bone [24]. The viability of osteocytes plays a significant role in maintaining bone homeostasis as cell death has been associated with pathological conditions such as osteoporosis and osteoarthritis [24], [25].

Mature osteoblasts express high levels of NPP1 [26] and, consequently, most studies to date have focused on the role of this enzyme in bone mineralisation and/or osteoblast differentiation [20], [27], [28]. The aim of this investigation was to determine whether the actions of NPP1 affect osteocyte function and cortical bone structure. The ability of NPP1 to influence osteoclast formation and bone resorption was also examined.

## Methods

### Reagents

All tissue culture and molecular biology reagents were purchased from Life Technologies (Paisley, UK) unless stated otherwise. Chemical reagents were purchased from Sigma Aldrich (Poole, Dorset, UK).

### *Enpp1^−/−^* mice

The generation and characterisation of *Enpp1*^−/−^ mice, which are on a 129Sv/TerJ genetic background, have previously been described [29]. All animal experiments were approved by UCL's animal users committee and animals were maintained in accordance with UK Home Office guidelines for the care and use of laboratory animals.

### Microcomputed tomographic (μCT) analysis of *Enpp1^−/−^* mice

The humerus, head, knees, spine, trachea and paws were isolated from 8, 15 and 22-week old *Enpp1*^−/−^ and wild-type (*Enpp1^+/+^*) mice and fixed in 10% neutral buffered formalin for 24 h. Samples were then washed in phosphate buffered saline and stored in 70% ethanol until scanning. All scans were performed using a Skyscan 1172 μCT scanner (Bruker, Kontich, Belgium). The humerus bones were scanned at 50 kV and 20 μA, using a 0.25 mm aluminium filter and a resolution of 0.9 μm. All other tissues were scanned using an image pixel size of between 5–10 μm and a 0.5 mm aluminium filter. To analyse the cortical bone of the humerus, a 0.25 mm region of interest was selected 0.5 mm below the deltoid tuberosity. The images were reconstructed using the Skyscan Nrecon software and analysed using the Skyscan CTan program.

### Histology

Histological analysis was performed on the trachea, ears and whisker follicles of *Enpp1^+/+^* and *Enpp1*^−/−^ mice. Tissues were fixed in 10% neutral buffered formalin and infiltrated with paraffin wax using an automated tissue processor (Leica Microsystems, Wetzlar, Germany). Samples were manually set within paraffin wax blocks before being cut into 3 μm sections and mounted onto slides. Before staining, the samples were de-paraffinised using xylene, then rehydrated through a series of decreasing ethanol solutions and finally water. Slides were stained for calcium using alizarin red (1% w/v), washed three times in deionised water and counter-stained with fast green (1% w/v). After staining, the samples were covered with a glass cover slip and imaged using a Nanozoomer slide imaging system (Hamamatsu, Hamamatsu City, Japan).

### Scanning electron microscopy

The femurs were dissected from 15 and 22-week old *Enpp1^+/+^* and *Enpp1*^−/−^ mice and cut along the longitudinal axis using a Buehler Isomet saw (Düsseldorf, Germany). The bone marrow, and any remaining soft tissue, was digested away in a 6% Tergazyme solution, pH 8.0 (Alconox, White Plains, NY, USA), for three weeks. The bones were dehydrated (50% ethanol, 2 h; 70% ethanol, 2 h; 100% ethanol, overnight) before being left to air dry. Images from the 15 and 22-week old animals were taken using a Zeiss EVO MA10 scanning electron microscope (SEM) (Zeiss, Oberkochen, Germany) at Queen Mary, University of London or a JEOL 7401 SEM (JEOL, Tokyo, Japan) at UCL, respectively. Analysis of SEM images (Image J, NIH, USA) was used to determine the number and size of blood vessel channels and osteocyte lacunae. A 2.0 mm region of interest located 4 mm below the growth plate on the endosteal surface of the cortical bone was selected for image analysis.

### Mouse osteocyte-like and osteoblast cell culture

Primary mouse osteocytes were obtained from the long bones of 15-week old *Enpp1^+/+^* and *Enpp1*^−/−^ using the methods described by Stern and colleagues [30]. Following isolation, cells were seeded onto collagen coated 6-well trays at a density of 2 × 10^5^ cells per well in αMEM. Half media changes were performed on every third day; the cells were used for experimental procedures on day 7. The number of cells present was counted manually using light microscopy.

Mouse osteoblasts were obtained from the calvaria of neonatal *Enpp1^+/+^* and *Enpp1*^−/−^ mice by trypsin/collagenase digestion as described previously [31]. Cells were cultured for up to 28 days in αMEM supplemented with 50 μg/ml ascorbate and 2 mM β-glycerophosphate.

### Total RNA extraction and complimentary DNA strand synthesis

Osteoclasts were cultured on dentine discs for up to 10 days before RNA was extracted using TRIzol® reagent according to the manufacturer's instructions. RNA was extracted from osteoblasts and osteocytes on day 21 and day 7 of culture, respectively. Before first stand complementary DNA (cDNA) synthesis extracted RNA was treated with RNase-free DNase I (35 U/ml) (Promega, Southampton, UK) for 30 min at 37 °C. The reaction was terminated by heat inactivation at 65 °C for 10 min. Total RNA was quantified spectrophotometrically by measuring absorbance at 260 nm. For each sample, 0.5 μg of DNase-treated total RNA was used as a template for first strand cDNA synthesis in a 20 μl reaction also containing 0.5 μg oligo dT, 3 mM MgCl_2_, 0.5 mM dNTPs, 20 U recombinant RNasin® ribonuclease inhibitor, ImProm-II® 5 × reaction buffer and 200 U ImProm-II reverse transcriptase. The reaction mix was annealed for 5 min at 25 °C, followed by extension at 42 °C for 60 min and inactivation at 70 °C for 15 min. cDNA was stored at − 20 °C until amplification by RT-PCR or qRT-PCR.

### RT-PCR

Osteoclast and osteocyte-derived cDNA was amplified by PCR in 25 μl reactions containing ~ 0.5 μg cDNA, 0.2 mM dNTP, 1.5 mM MgCl_2_, 0.2 μM of both sense and anti-sense primer, 1 U Taq DNA polymerase in thermophilic DNA polymerase 10 × buffer. PCR was performed according to the manufacturer's instructions for between 30–40 cycles of amplification. Denaturation at 95 °C for 30 s, annealing for 30 s at a primer specific temperature and extension at 72 °C for 45 s took place within each cycle, followed by reaction termination at 72 °C for 5 min. For analysis, PCR products were loaded on to 1% agarose gels containing 0.3 μg/ml ethidium bromide. Gels were run at ~ 80 mA and the DNA position visualised by exposure to UV light. To account for differences in original cell number and cDNA quality, osteoclast samples were normalised against mRNA for GAPDH. All reactions were carried out in triplicate using cDNAs derived from 3 different osteoclast/osteocyte cultures. Primer sequences are shown in Table 1. The RT-PCR positive control used samples from mouse brain.

**Table 1 Primer sequences for RT-PCR.** 

Mus musculus gene	Sense 5′–3′	Anti-sense 5′–3′
*GAPDH*	CTCACTCAAGATTGTCAGCA	GTCATCATACTTGGCAGGTT
*Enpp1*	ACAGCTTAATCTGACCACAG	GATCCTGGTACAGACAGTTG
*Enpp2*	GTATGACCCTGTCTTTGATG	GAAAGCCACTGAAGGATAGT
*Enpp3*	CTGCTGACTGTGGTTTTACT	CTGTGGTAAAGGAGACAGTG
*NTPdase1*	CTTTGGCGCTTTGGATCTCG	TCTGGTGGCACTGTTCGTAG
*NTPdase2*	CTGGAGGCAGTGACACAGAC	TGGGTGGAGTAGCCCTTTGG
*NTPdase3*	GTGAGCATTGTGGTACTTGT	TGACCACTCCTGTGTTATTC
*ANK*	CAGTTTCCTGGTGGGATGTG	TTGATGTGGGCTGAGGTG

*GAPDH* = glyceraldehyde-3-phosphate dehydrogenase, *Enpp* = ecto-nucleotide pyrophosphatase/phosphodiesterase, *NTPdase* = ecto-nucleoside triphosphate diphosphohydrolase, *ANK* = progressive ankylosis gene.

### Quantitative real-time PCR (qRT-PCR)

Osteoblast and osteocyte cDNAs were amplified using specific QuantiTect primers (β-actin, sclerostin) and QuantiTect SYBR® Green (Qiagen Ltd, Crawley UK). qRT-PCR (Chromo4, Biorad Laboratories Ltd, Hemel Hempstead, UK) was performed according to manufacturer's instructions with an initial activation step (94 °C for 15 min) followed by 40 cycles of denaturation (95 °C for 10 s) and detection (60 °C for 30 s). Gene expression was investigated in osteocytes and mature, bone forming osteoblasts. Data was analysed using the Pfaffl method [32] and is shown as relative expression of sclerostin in *Enpp1^+/+^* and *Enpp1*^−/−^ cells. All reactions were carried out in triplicate using RNAs derived from 3 different osteoblast/osteocyte cultures.

### Measurement of serum sclerostin

Blood was collected from 8, 15 and 22-week old *Enpp1*^−/−^ and *Enpp1^+/+^* mice by cardiac puncture immediately after termination. Following clotting, samples were centrifuged at 500 *g* and the serum was retained and frozen until analysis. Serum sclerostin was measured using a commercially available ELISA kit (R&D systems, Abingdon, UK), as per the manufacturer's instructions. It is unknown which of the many forms of secreted sclerostin [33] are detected by this kit.

### Measurement of extracellular ATP

Prior to measurement of ATP levels, culture medium was removed, cell layers were washed and cells were incubated with serum-free DMEM (1 ml/well) for 1 h. Extracellular ATP release was measured luminetrically using the *luciferin–luciferase* assay as previously reported [34]. Cell number was determined using the CytoTox 96® non-radioactive cytotoxicity assay (Promega, Southampton, UK).

### Osteoclast formation assay

Osteoclasts were generated from the bone marrow of 8 or 15-week old *Enpp1^+/+^* or *Enpp1^−/−^* mice as previously reported [35]. Briefly, cells were plated onto 5 mm diameter dentine discs (10^6^ cells) in αMEM supplemented with 100 nM PGE_2_, 200 ng/ml M-CSF and 3 ng/ml receptor activator of nuclear factor _Κ_B (RANKL; R&D Systems Europe Ltd, Abingdon, UK). After 24 h, discs containing adherent osteoclast precursors were transferred to 6 well trays (4 discs/well in 4 ml medium) for a further 6 days at 37 °C in 5% CO_2_/95% atmospheric air. Culture medium was acidified to pH ~ 7.0 by the addition 10 meq/l H^+^ (as HCL) on day 7 to activate osteoclastic resorption of dentine [35], [36], [37], [38]. Culture medium pH, pCO_2_ and PO_2_ were monitored throughout using a blood gas analyser (ABL 705, Radiometer, Copenhagen, Denmark). Osteoclasts were fixed in 2% glutaraldehyde and stained to demonstrate tartrate-resistant acid phosphatase (TRAP). Osteoclasts were defined as TRAP-positive cells with 2 or more nuclei and/or clear evidence of resorption pit formation. Osteoclast number and the plan surface area of resorption pits were measured as described previously [35].

### Total cellular NPP activity measurement

The assay used to measure total cellular NPP activity was based on the method originally described by Razzell and Khorana [39]. Briefly, cells were lysed in a buffer containing 1% triton × 100 in a 0.2 M Tris base with 1.6 mM MgCl_2_, pH 8.1. Following centrifugation at 500 *g* the NPP activity of collected supernatants was measured using 5 mM p-nitrophenyl-thymidine 5′-monophosphate as substrate. Total protein concentration in cell l lysates was measured using the Bradford method.

### Statistics

Statistical comparisons were made using a *t*-test or one-way analysis of variance (ANOVA) and adjusted using the Bonferroni method [40], [41]. Calculations were performed using In Stat 3 (GraphPad, San Diego, CA). All data are presented as means ± SEM for between 6–12 replicates. Results are representative of experiments performed at least three times, unless otherwise stated.

## Results

### Ectopic mineralisation in *Enpp1^−/−^* mice

**Fig. 1:**
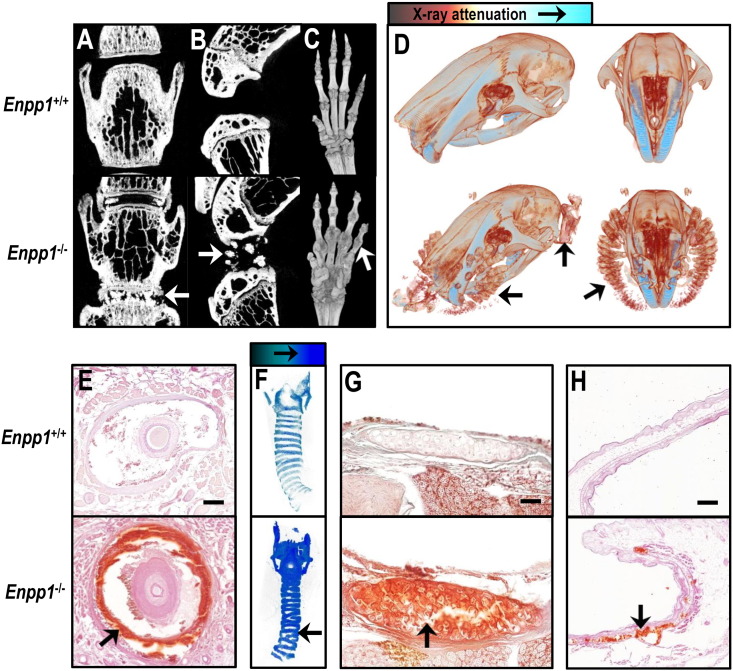
μCT and histology show that Enpp1^−/−^ mice have nonspecific tissue mineralisation. μCT imaging was used to visualise the spine (A), knee joints (B) and paws (C) of *Enpp1^+/+^* and *Enpp1*^−/−^ mice. Dystrophic mineralisation was seen between the vertebrae, within the knee joint, and surrounding the joints of the paw. (D) μCT scanning of the heads from *Enpp1^+/+^* and *Enpp1*^−/−^ mice with all the soft tissue attached indicated that whisker follicles and ear pinna were mineralised. (E) Alizarin red staining of histological sections showed that the collagen ring that sounds the whisker was mineralised in *Enpp1*^−/−^ mice. (F) μCT scanning and (G) histology revealed mineralisation in the cartilage rings of the trachea in *Enpp1*^−/−^ animals. (H) Sectioning and alizarin red staining of the ear also revealed mineralisation within this tissue in *Enpp1*^−/−^ mice. The representative images shown are from 15 (A–C) and 22-week old (D–H) animals. Scale bars: E = 100 μm, G = 150 μm, H = 50 μm.

Previous studies have reported aberrant mineral deposition in *Enpp1*^−/−^ mice [4], [20], [27]. Here, we used μCT imaging to visualise this ectopic mineralisation at high resolution. *Enpp1^−/−^* mice displayed mineralisation between the vertebrae (Fig. 1A) and of the ligaments within the knee joint (Fig. 1B). Within the paws, there was mineralisation of the joint capsules and the metatarsals and phalanges were partially fused together (Fig. 1C).

Interestingly, we also observed mineral deposits in several collagen rich soft tissues including the whisker follicles, trachea and ear pinna of *Enpp1^−/−^* mice (Figs. 1D–H). The mineralisation of the whisker follicles was sub-dermal, not visible to the naked eye, nor easily detectable by touch. To ensure that the mineralisation of the vibrissae observed by μCT was not an artefact of the scanning, histology was performed on the whisker follicles of *Enpp1^+/+^* and *Enpp1*^−/−^ mice. Alizarin red staining showed that the collagenous sheath surrounding the whisker follicles in *Enpp1*^−/−^ mice contained calcium mineral. No other mineralisation was detected in this tissue and the smaller hair follicles, which are not surrounded by collagen, were not mineralised (Fig. 1E). μCT analysis and histology also demonstrated mineralisation within the cartilage rings of the trachea (Figs. 1F & G) and the ear pinna of *Enpp1*^−/−^ mice (Figs. 1D & H).

In all cases, soft tissue mineralisation was evident from 8 weeks and worsened with age; the representative images shown are from 15 (Figs. 1A–C) and 22-week old (Figs. 1D–H) animals.

### *Enpp1^−/−^* mice have thinner and less porous cortical bone with a bigger bone marrow cavity

**Fig. 2:**
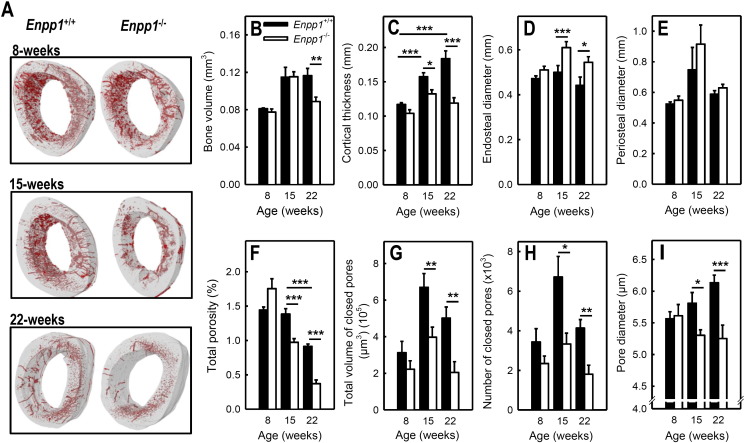
Enpp1^−/−^ mice display significant changes in the cortical bone. Humerus bones from 8, 15 and 22-week old mice were scanned by μCT at a resolution of 0.9 μm. Measurements were performed on a region of interest 0.25 mm in length, 0.5 mm below the deltoid tuberosity. (A) Representative images of the cortical bone from *Enpp1^+/+^* and *Enpp1*^−/−^ mice. The grey regions represent the bone and the red areas the spaces within the bone. No differences in the cortical bone were observed in 8-week old animals. (B) Cortical bone volume was decreased by ≤ 24% in *Enpp1*^−/−^ mice. (C) Cortical thickness was reduced 16% and 35% in 15 and 22-week old *Enpp1*^−/−^ mice, respectively. (D) The endosteal diameter was increased up to 23% in *Enpp1*^−/−^ animals. (E) The periosteal diameter was unchanged by NPP1 deletion. (F) Total porosity was decreased 30% and 60% in 15 and 22-week old *Enpp1*^−/−^ mice, respectively. (G) The total volume of closed pores was reduced up to 55% in *Enpp1*^−/−^ animals. (H) The number of closed pores was decreased 40% and 59% at 15 and 22-weeks, respectively. (I) Pore diameter was reduced up to 15% in *Enpp1*^−/−^ mice. Values are means ± SEM (n = 5–10), *** = p < 0.001, ** = p < 0.01, * = p < 0.05.

This study used high resolution μCT to examine in detail how NPP1 deletion affects cortical bone structure. Representative images of the cortical bone at 8, 15 and 22-weeks are shown in Fig. 2A. No differences in any of the cortical bone parameters measured were observed in the 8-week animals (Fig. 2). Cortical bone volume was decreased up to 28% in *Enpp1^−/−^* mice (Fig. 2B). This was accompanied by 16% and 35% reduction in cortical thickness in 15 and 22-week old *Enpp1*^−/−^ animals, respectively (Fig. 2C). *Enpp1^+/+^* mice displayed increasing cortical thickness with age (57% higher at 22-weeks compared to 8-weeks); this age-related increase in cortical thickness did not occur in *Enpp1*^−/−^ mice (Fig. 2C). The endosteal diameter, which represents the diameter of the bone marrow cavity, was increased up to 23% in *Enpp1*^−/−^ mice (Fig. 2D). The periosteal diameter was unchanged by NPP1 deletion (Fig. 2E).

Total porosity represents all the space within the cortical bone that is not filled by bone mineral *e.g.* blood vessels channels or osteocyte lacunae. We found that total porosity was decreased 30% and 60% in 15 and 22-week old *Enpp1*^−/−^ mice, respectively (Figs. 2A & F). The total volume of closed pores, which equates to the spaces totally enclosed by mineral, was reduced up to 55% in *Enpp1*^−/−^ animals (Fig. 2G). The number of closed pores was decreased 40% and 59% at 15 and 22-weeks, respectively (Fig. 2H). Pore diameter was reduced up to 15% in *Enpp1*^−/−^ mice (Fig. 2I).

Differences in the total x-ray attenuation of the μCT scans can indicate whether a tissue is hypermineralised (*i.e.* the matrix is overly mineralised). Analysis of the x-ray attenuation within the cortical bone revealed no differences between the *Enpp1^+/+^* and *Enpp1*^−/−^ mice at any age (not shown).

### Reduced size of vascular channels and osteocyte lacunae in *Enpp1^−/−^* mice

**Fig. 3:**
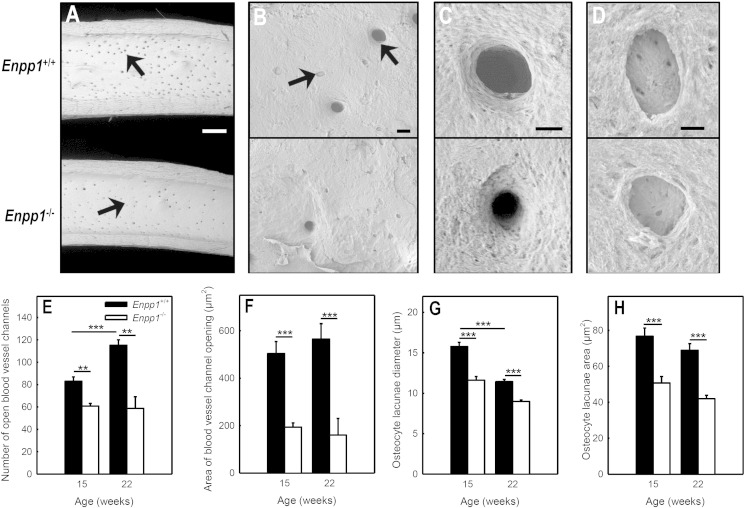
Reduced vascular channels and smaller osteocyte lacunae in Enpp1^−/−^ mice. Representative SEM images of the femoral endosteal surface from *Enpp1^+/+^* and *Enpp1*^−/−^ mice. (A) Vascular channels are reduced in size and number in *Enpp1*^−/−^ animals. Higher magnification images highlight the decreased size of the (B, C) blood vessel channels and (B, D) osteocyte lacunae in *Enpp1*^−/−^ mice. Scale bars: A = 0.5 mm, B = 20 μm, C = 12 μm, D = 5 μm. (E, F) The number and size of blood vessel channels on the endosteal surface of the bone were reduced up to 50% and 70%, respectively, in *Enpp1*^−/−^animals. (G, H) Osteocyte lacunae diameter and surface area were reduced ≤ 25% and ≤ 39%, respectively, in *Enpp1*^−/−^ mice. Quantitative analysis of blood vessel channels and osteocyte lacunae images based on n = 50 measurements per group; *** = p < 0.001, ** = p < 0.01.

Low power SEM analysis of the femur showed that the number and size of the vascular channels on the endosteal surface of the cortical bone was reduced up to 50% and 70%, respectively in *Enpp1*^−/−^ mice (Figs. 3A–C & E–F). SEM at higher power was then used to visualise osteocyte lacunae on cortical bone surfaces (Figs. 3B & D). Quantification of SEM images showed that the lacunae diameter was reduced up to 25% in *Enpp1*^−/−^ mice (Fig. 3F). The plan surface area of the lacunae was decreased 35% and 39% in 15 and 22-week old *Enpp1*^−/−^ animals (Fig. 3G). It was also noted that in *Enpp1*^+/+^ animals there was a reduction in the size of the osteocyte lacunae as the animals aged (Fig. 3G).

### Expression of NPP1 by mouse osteocyte-like cells

**Fig. 4:**
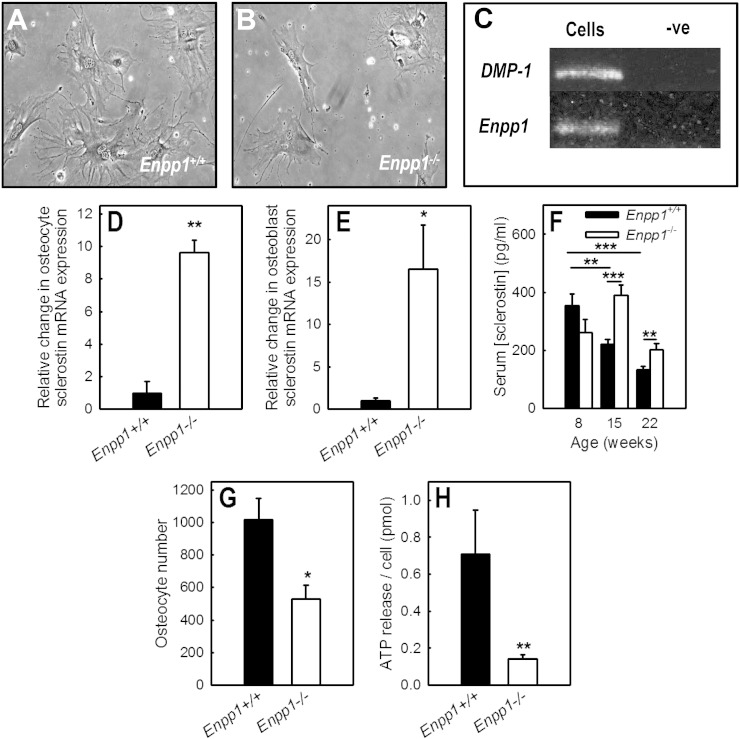
The effect of NPP1 deletion on osteocyte number, ATP release and serum sclerostin levels. (A, B) Osteocyte-like cells extracted from the long bones of 15-week old mice displayed dendritic processes characteristic of these cells. (C) RT-PCR showed mRNA expression of *DMP-1* and *Enpp1* in *Enpp1^+/+^* osteocyte-like cells. qRT-PCR revealed that sclerostin mRNA expression was increased. (D) ~ 9-fold increase osteocytes and (E) ~ 16-fold in mature, bone forming osteoblasts. (F) Sclerostin levels were increased 75% and 52% in 15 and 22-week old *Enpp1^−/−^* mice, respectively. (D) The number of osteocyte-like cells was reduced 48% in *Enpp1^−/−^*cultures. (E) *Enpp1^−/−^* cells displayed an 80% decrease in constitutive ATP release. Values are means ± SEM (n = 5–10), *** = p < 0.001, ** = p < 0.01, * = p < 0.05.

Mouse osteocyte-like cells extracted from the long bones of 15-week old *Enpp1^−/−^* and *Enpp1^+/+^* mice displayed the characteristic dendritic processes of osteocytes (Figs. 4A & B). RT-PCR showed that *Enpp1^+/+^* osteocyte-like cells express mRNA for the osteocyte specific gene, *DMP1*, and for *Enpp1* (Fig. 4C).

### Increased sclerostin levels in *Enpp1^−/−^* mice

Sclerostin mRNA expression was increased ~ 9-fold in osteocytes (Fig. 4D) and ~ 16-fold in mature, bone forming osteoblasts (Fig. 4E) derived from *Enpp1^−/−^* mice. No differences in the serum concentration of sclerostin were seen in 8-week old animals. However, sclerostin levels were increased 75% and 52% in 15 and 22-week old *Enpp1^−/−^* mice, respectively (Fig. 4F). In *Enpp1^+/+^* animals, ageing was associated with a 62% reduction in serum sclerostin levels; this decrease was not seen in *Enpp1^−/−^* mice.

### Reduced number and ATP release in cultures of *Enpp1^−/−^* osteocyte-like cells

Osteocyte-like cells extracted from the bones of *Enpp1^−/−^* and *Enpp1^+/+^* mice were cultured for 7 days. The number of osteocyte-like cells was reduced by 48% in *Enpp1^−/−^* cultures (Fig. 4G). Constitutive ATP release from *Enpp1^−/−^* osteocyte-like cells into the culture medium was decreased by 80% compared to *Enpp1^+/+^* cells (Fig. 4H).

### Osteoclasts expressed mRNA for NPP1 and other ecto-nucleotidases

**Fig. 5:**
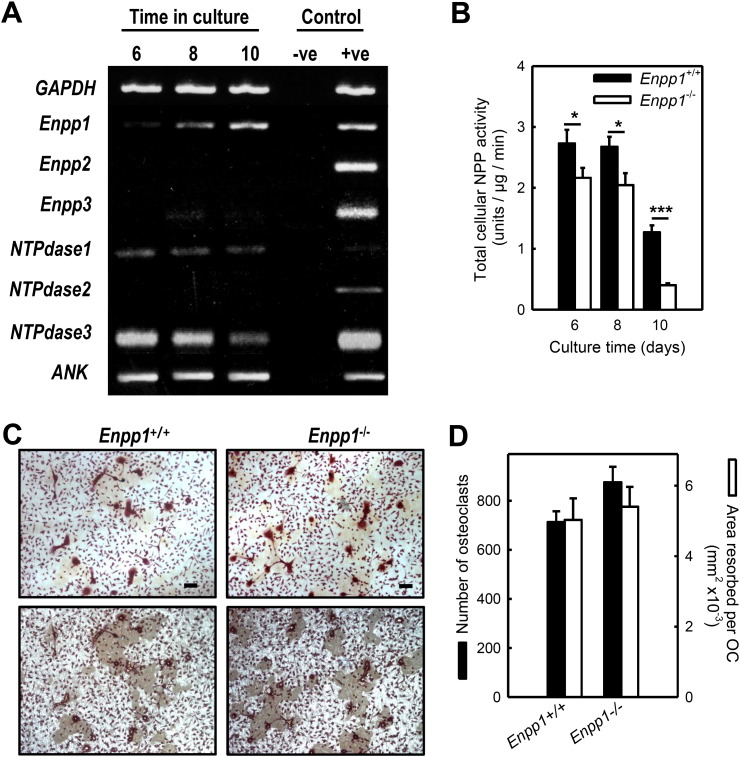
NPP1 deletion does not affect the formation or resorptive activity of osteoclasts. (A) RT-PCR analysis was performed on osteoclast precursors (day 3), early osteoclasts (day 6), mature osteoclasts (day 8) and mature resorbing osteoclasts (day 10), Expression of *Enpp1*, *Enpp3*, *NTPdase1*, *NTPdase3* and *Ank* mRNA was detected from day 6 onwards (day 3 not shown). Expression of *Enpp2* and *NTPdase2* mRNAs was not detected. RT-PCR positive control = mouse brain. (B) Osteoclasts exhibit functional NPP activity, which decreases as osteoclastogenesis progresses. *Enpp1^−/−^* osteoclasts displayed up to a 70% reduction in total NPP activity. (C) Representative transmitted (top) and reflective (bottom) light microscopy images of *Enpp1^+/+^* and *Enpp1*^−/−^ osteoclasts cultured on dentine. Scale bar = 50 μm. (D) No differences in osteoclast formation or resorptive activity was seen in *Enpp1^−/−^* osteoclasts. Values are means ± SEM (n = 8–10), *** = p < 0.001, * = p < 0.05.

RT-PCR analysis was performed on osteoclast precursors (day 3), early osteoclasts (day 6), mature osteoclasts (day 8) and mature resorbing osteoclasts (day 10) to investigate the expression of ecto-nucleotides in these cells. We found that osteoclasts express mRNAs for *Enpp1*, *Enpp3*, *NTPdase1*, *NTPdase3* and the transport protein, *Ank*, from day 6 onwards; mRNA expression of *Enpp2* and *NTPdase 2* was not detected (Fig. 5A). *Enpp1* mRNA expression increased as osteoclastogenesis progressed with peak levels in mature, resorbing cells. In contrast, mRNA levels of *Enpp3* and *NTPdase 3* were lower in mature osteoclasts. Expression of *Ank* and *NTPdase 1* mRNA remained relatively constant (Fig. 5A).

### Osteoclasts display NPP activity

Total cellular NPP activity was measured in osteoclasts at all stages of differentiation. We found that osteoclasts exhibited functional NPP activity, which decreased as osteoclastogenesis progressed (Fig. 5B) *Enpp1^−/−^* osteoclasts displayed up to a 70% reduction in total NPP activity (Fig. 5B).

### Osteoclast formation and activity is unaffected by NPP1 deletion

Osteoclasts were generated from 8 and 15-week old *Enpp1^−/−^* and *Enpp1^+/+^* mice and cultured on dentine discs for 9 days. Representative transmitted and reflective light microscopy images of the osteoclasts formed from these animals are shown in Fig. 5C. No differences in osteoclast formation or resorptive activity were seen in *Enpp1^−/−^* osteoclasts from 8-week (Fig. 5D) and 15-week old (not shown) animals.

## Discussion

The role of NPP1 in tissue mineralisation has been widely studied using rodent models. In particular, the global ablation of *Enpp1^−/−^* function has provided considerable information about the actions of NPP1 in different tissues (see review by Mackenzie et al. 2012 [18]). Within bone, the effects of NPP1 on osteoblast differentiation and function have been well documented [20], [27], [28], yet information regarding its role in osteocytes and osteoclasts is limited. This study demonstrated for the first time that NPP1 is expressed by both cell types. Using *Enpp1^−/−^* mice we identified a key role for NPP1 in maintaining the size of osteocyte lacunae.

Earlier investigations reported that *Enpp1^−/−^* mice exhibit aberrant mineralisation of the aorta, joints, spine and cartilage [4], [19], [20], [21]. During our μCT analysis of these mice we also observed extensive ectopic calcification in the above tissues. We additionally saw mineralisation in several soft tissues where it had not previously been detected. The whisker follicles, ear pinna and trachea all contain type I or type II collagen which could act as a site for mineral deposition. The mineralisation of these tissues in knockout mice suggests that, under normal conditions, production of PP_i_ from ATP by NPP1 is important in preventing calcification.

The initial studies of *Enpp1^−/−^* mice showed that these animals had reduced bone mineral content (BMC) in the long bones [27]. Subsequent, more detailed analysis by Mackenzie et al. [20] showed that *Enpp1^−/−^* mice display reduced trabecular bone volume, thickness and number as well as decreased cortical thickness and porosity. In agreement, we also observed age-related decreases in cortical bone volume, thickness and porosity. Additionally, we found that whilst the periosteal diameter was unchanged the endosteal diameter was increased making the cortical bone in *Enpp1^−/−^* mice thinner and less porous with a larger marrow space. Taken together, these studies show that the reduced BMC of the long bones originally identified by Anderson et al. [27] involves decreased bone in both the trabecular and cortical compartments.

Cortical porosity, as measured using μCT, is a measurement of all of the spaces within the bone including vascular channels, osteocyte lacunae or microcracks. The μCT analysis performed by Mackenzie et al. [20] used a resolution of 5 μm, which although adequate for determining overall changes in cortical bone volume and porosity, was not sufficient to provide detailed information about the osteocyte lacunae. Here, we used higher resolution μCT (0.9 μm) and SEM to examine whether the changes in cortical porosity could reflect alterations in the size of osteocyte lacunae in *Enpp1^−/−^* mice. We found that the number, volume and diameter of the closed pores were reduced in *Enpp1^−/−^* animals; this was accompanied by a decrease in the number of osteocytes isolated from the long bones. Subsequent analysis of SEM images also showed a reduction in lacunar diameter and area. The most likely explanation for the decreased osteocyte lacunar size in *Enpp1^−/−^* mice is a localised increase in the amount of matrix mineralisation, which could have a negative impact on osteocyte function and survival. Further studies will be needed to assess the effect of NPP1 deletion on osteocyte numbers *in situ* and on the fine canaliculae that interconnect these cells. Previously, increased mineralisation of the osteocyte lacunae has been linked with ageing [42], osteoporosis and osteoarthritis [25].

Cultured MLO-Y4 osteocyte-like cells have been reported to release ATP constitutively [43], [44]. The present work shows that primary osteocyte-like cells from mouse long bones also release ATP and, moreover, express mRNA for NPP1. This suggests that osteocytes are capable of hydrolysing ATP to generate the mineralisation inhibitor, PP_i_, in their extracellular environment. Earlier work has shown that extracellular PP_i_ levels are reduced in the culture medium of *Enpp1^−/−^* osteoblasts [19]. Therefore, it is possible that the decreased lacunar size in *Enpp1^−/−^* mice is due to increased mineralisation that is the result, at least in part, of a lower concentration of ATP-derived PP_i_.

Despite exhibiting mineralisation of soft tissues and osteocyte lacunae, *Enpp1^−/−^* mice display a decreased trabecular and cortical bone volume [20]. It has been suggested that the reduced bone seen in *Enpp1^−/−^* animals occurs because there is insufficient PP_i_ for TNAP to generate the P_i_ needed for normal bone formation [18]. However, the reduction in the size and number of the vascular channels within the cortical bone of *Enpp1^−/−^* mice could provide a further pathophysiological mechanism for the decreased bone mass in these animals. A decreased vascular supply to the bones, perhaps exacerbated by the aortic calcification previously described in *Enpp1^−/−^* mice [18], [45], would result in hypoxia and acidosis, which are well known to increase osteoclast formation and resorption [36], [37], whilst also inhibiting bone mineralisation [46], [47], [48]. In support of this notion, we observed prominent fields of resorption pits on the endosteal surfaces of *Enpp1^−/−^* bones and *Enpp1*^−/−^ mice have increased levels of potassium ions, which can be a marker of acidosis, within the blood [20].

Sclerostin, which is secreted by osteocytes, inhibits Wnt signalling and negatively regulates bone formation [49]. We found that serum sclerostin levels were higher in *Enpp1^−/−^* mice and that sclerostin mRNA expression was strongly increased in *Enpp1^−/−^* osteocyte-like cells and mature, bone forming osteoblasts. Previous studies have shown that mechanical loading regulates sclerostin expression, with increased levels in unloaded bones [50], [51], [52], [53], [54]. *Enpp1^−/−^* mice display an unusual walking gait [17], [29], muscle damage [20] and mineralisation of the spine, knees and toes, which combined are likely to affect the mechanical loading of their bones. Thus, the elevated blood sclerostin in *Enpp1^−/−^* mice may be due not only to constitutively higher production by bone cells but also to decreased mechanical loading. These factors are consistent with the reduced bone mass observed in these animals. *Enpp1^−/−^* mice also display increased expression of FGF23, an osteocyte-derived phosphaturic hormone that can inhibit bone mineralisation [20]. Recently it was reported that sclerostin can indirectly regulate FGF23 expression since sclerostin knockout mice display reduced levels of serum FGF23 [55]. Therefore, the increased FGF23 levels in *Enpp1^−/−^* mice could be a consequence of the higher sclerostin levels.

This study demonstrated for the first time that osteoclasts express several ecto-nucleotidases including NPP1. Osteoclasts also exhibit significant NPP activity making them capable of contributing to extracellular PP_i_ levels. A significant portion of this NPP activity was due to NPP1 suggesting that, like osteoblasts, this is the pre-eminent NPP in osteoclasts. μCT analysis showed that whilst the endosteal diameter was increased in *Enpp1^−/−^* mice, the periosteal diameter was unchanged. This increase in the endosteal/periosteal ratio is indicative of increased osteoclast activity in the bone. In agreement, Mackenzie and colleagues saw histological and biochemical evidence of increased *in vivo* osteoclast activity in *Enpp1*^−/−^ mice [20]. Our current study found no differences in osteoclast formation and activity when *Enpp1^−/−^* osteoclasts were generated *in vitro* from bone marrow precursors. However, the SEM images shown in Fig. 3 do show an apparent increase in bone resorption on the endosteal surface of the cortical bone from *Enpp1*^−/−^ mice. The discrepancy between the *in vitro* and *in vivo* data suggests that any effects of NPP1 deletion on osteoclasts are mediated by other cells.

In summary, the work presented here shows that NPP1 plays a key role in regulating soft tissue and skeletal mineralisation. The ectopic mineralisation observed in several collagen rich soft tissues suggests that NPP1 expression is more widespread than previously reported. Our findings also suggest that expression of NPP1by osteocytes may be important in preventing the over-mineralisation of their lacunae (which could potentially compromise osteocyte function or survival). Finally, since age-related pathological calcification is a common in humans, the rapidly worsening phenotype of *Enpp1*^−/−^ mice may, in some respects, represent a useful model of accelerated ageing.
